# The Impact of Significant Other Expressed Emotion on Patient Outcomes in Chronic Fatigue Syndrome

**DOI:** 10.1037/hea0000086

**Published:** 2014-09

**Authors:** Rebecca Band, Christine Barrowclough, Alison Wearden

**Affiliations:** 1 School of Psychological Sciences & Manchester Centre for Health Psychology, University of Manchester, and Centre for Applications of Health Psychology, University of Southampton, United Kingdom; 2 School of Psychological Sciences, University of Manchester, United Kingdom

**Keywords:** chronic fatigue syndrome, significant others, expressed emotion, criticism

## Abstract

***Objective:*** Previous literature has identified the importance of interpersonal processes for patient outcomes in chronic fatigue syndrome/myalgic encephalomyelitis (CFS/ME), particularly in the context of significant other relationships. The current study investigated expressed emotion (EE), examining the independent effects of critical comments and emotional overinvolvement (EOI) in association with patient outcomes. ***Method:*** Fifty-five patients with CFS/ME and their significant others were recruited from specialist CFS/ME services. Significant other EE status was coded from a modified Camberwell Family Interview. Patient outcomes (fatigue severity, disability, and depression) were derived from questionnaire measures. Forty-four patients (80%) completed follow-up questionnaires 6-months after recruitment. ***Results:*** Significant other high-EE categorized by both high levels of critical comments and high EOI was predictive of worse fatigue severity at follow-up. High-critical EE was associated with higher levels of patient depressive symptoms longitudinally; depressive symptoms were observed to mediate the relationship between high critical comments and fatigue severity reported at follow-up. There were higher rates of high-EE in parents than in partners, and this was because of higher rates of EOI in parents. ***Conclusions:*** Patients with high-EE significant others demonstrated poorer outcomes at follow-up compared with patients in low-EE dyads. One mechanism for this appears to be as a result of increased patient depression. Future research should seek to further clarify whether the role of interpersonal processes in CFS/ME differs across different patient-significant other relationships. The development of significant other-focused treatment interventions may be particularly beneficial for both patients and significant others.

Chronic fatigue syndrome (or myalgic encephalomyelitis; CFS/ME) is a complex condition characterized by severe, persistent fatigue, together with other symptoms including headaches, sleep disturbances, cognitive complaints, and muscular and joint pain (Fukuda et al., 1994). The estimated prevalence of the condition has varied widely; ranging from ∼0.2–2.6% within adult populations in various settings in the United States and United Kingdom (Prins, van der Meer, & Bleijenberg, 2006). A diagnosis of CFS/ME is made when other potential medical explanations for the fatigue and other symptoms have been ruled out; it is therefore, a diagnosis of exclusion (Fukuda et al., 1994). However, explanatory models propose that CFS/ME represents a state of physiological dysregulation in which the maintenance of established symptoms may be influenced by multiple interacting factors, such as patient symptom preoccupation, beliefs, and behaviors in response to the condition (Deary, Chalder, & Sharpe, 2007; Surawy, Hackmann, Hawton, & Sharpe, 1995).

Social factors, such as medical uncertainty and illness legitimacy may also contribute toward the maintenance of CFS/ME (Deary et al., 2007). The challenges associated with negotiating a diagnosis (Dickson, Knussen, & Flowers, 2007; Larun & Malterud, 2007) have been proposed to increase the importance of significant others’ views in informing patient understanding of their condition (Cordingley, Wearden, Appleby, & Fisher, 2001); both patients and significant others report collaborative explanation seeking with respect to the condition (Brooks, King, & Wearden, 2013). Furthermore, in association with high levels of patient disability, reduced social contact, and shifting roles within the family (Ax, Gregg, & Jones, 2002; McCrone, Darbishire, Ridsdale, & Seed, 2003), the functional role of significant others in providing emotional or instrumental support (Melamed, 2003) may become more important over time. The lack of established biomedical markers for the condition often results in delegitimizing experiences for patients from both health care professionals and significant others (Dickson et al., 2007). Patients report these delegitimizing interactions with significant others as the most difficult to deal with and they are associated with patient accounts of poor coping and feeling unsupported (Dickson et al., 2007). Therefore, there is good reason to suggest that it would be beneficial to examine the role of interpersonal factors in the context of ongoing CFS/ME (Blazquez & Alegre, 2013; Melamed, 2003).

Empirical studies examining significant other behaviors in response to patient symptom experiences, such as fatigue or pain, have noted associations with patient illness outcomes, including symptom severity and psychological wellbeing. In particular, solicitous responses, for example, comforting the patient or helping with practical tasks (Kerns & Rosenberg, 1995), have been associated with increased fatigue severity and disability (Romano, Jensen, Schmaling, Hops, & Buchwald, 2009; Schmaling, Smith, & Buchwald, 2000). In comparison, negative responses, for example, expressing anger or frustration at the patient (Kerns & Rosenberg, 1995) have been associated with poorer psychological outcomes and increased depression (Romano et al., 2009). Increased emotional difficulties in CFS/ME, particularly depression, have been noted to predict poorer long-term patient outcomes and responses to therapeutic interventions (Bentall, Powell, Nye, & Edwards, 2002; Wearden, Dunn, Dowrick, & Morriss, 2012). Therefore, as negative and solicitous significant other responses appear to impact upon patient outcomes in different ways, these findings suggest that there are potentially two interpersonal processes that that may be important in symptom perpetuation, and provide the rationale for further exploration of significant other responses in CFS/ME.

A well-established framework for empirically examining significant other factors in association with patient outcomes is the multicomponent expressed emotion (EE) construct (Vaughn & Leff, 1976). EE provides a measure of several aspects of the patient-significant other relationship; it is thought to represent the affective quality of the relationship, related to significant other distress and burden (Barrowclough & Parle, 1997; Tarrier et al., 2002), and has been shown to reflect typical patterns of reciprocal interaction within the dyad (Hooley, 1986, 2007; Miklowitz, Goldstein, & Nuechterlein, 1995). Significant others are conventionally conceptualized as high- or low-EE on the basis of ratings of key components of the construct; critical comments, emotional overinvolvement (EOI), and hostility. Hostility is often considered as an extreme form of criticism (Wearden, Tarrier, Barrowclough, Zastowny, & Rahill, 2000) and is rarely observed in the absence of highly critical attitudes (Leff & Vaughn, 1985). Ratings of EE are made on the basis of evidence for these constructs during the semistructured Camberwell Family Interview (CFI; Vaughn & Leff, 1976). Alongside consideration of the content of speech, which may include evidence of significant other beliefs, attitudes, and responses toward the patient, ratings are made on the basis of the tone of speech and significant other behaviors at interview, such as dramatization or emotional display. A robust association has been documented between the presence of a high-EE relative and poorer patient illness and treatment outcomes, particularly across psychiatric conditions, but also among patients who are experiencing physical health problems (Bebbington & Kuipers, 1994; Butzlaff & Hooley, 1998; Hooley, 2007; Tarrier, Sommerfield, & Pilgrim, 1999; Wearden, Tarrier, Barrowclough, et al., 2000; Wearden, Tarrier, & Davies, 2000).

The assumption that significant other EE may drive significant other behavioral responses toward the patient has received some support in the form of observational data (Hooley, 1986; Miklowitz et al., 1995). Much of the previous EE research has focused upon criticism, although it has been noted that significant other emotional and behavioral responses are likely to be different when arising as a result of high EOI (Vasconcelos e Sa, Wearden, & Barrowclough, 2013; Wearden, Tarrier, Barrowclough, et al., 2000). Previous research on significant other behavioral responses in CFS/ME suggests that there are two distinct response styles (i.e., solicitous and negative) that are important for patient outcomes (Romano et al., 2009; Schmaling et al., 2000); the constituent behaviors for these response styles (Kerns & Rosenberg, 1995) overlap with the responses that would be associated with ratings on the EOI and critical comments EE subscales. For example, solicitous behaviors such as showing high levels of concern for the patient or engaging in tasks on the patients’ behalf would count toward the rating of EOI. Additionally, behaviors that are reportedly associated with negative responses, such as expressing frustration, overlap with the EE construct of criticism. Therefore, it was hypothesized that solicitous responses may reflect behaviors driven by high levels of EOI, while negative responses may be associated with high levels of criticism. No previous study has considered the role of significant other EE as a framework for examining interpersonal processes in CFS/ME. Therefore, the aim of the current study was to extend the previous literature by being the first to examine how significant other EE is associated with patient outcomes in a CFS/ME population, and to assess these associations both cross-sectionally and longitudinally over 6 months. The proposed relationships between the behavioral responses previously associated with CFS/ME outcomes and the principle EE subscales were utilized to guide study hypotheses.

Within the wider CFS/ME literature, research examining significant other factors have tended to focus exclusively upon romantic partners or spouses. However, within EE research, significant others have typically been defined as the person with the most daily involvement in the patients’ illness management (MacCarthy, Hemsley, Shrank-Fernandez, Kuipers, & Katz, 1986), often with a minimum limit of weekly contact specified (Onwumere et al., 2008); consequently, a variety of significant other relationship types have been examined, often reflecting the characteristics of the disorder under examination. These relationship types are seldom distinguished between when examining the predictive validity of EE, although there are some findings of note in the literature. Investigations of the role of EE in depression, have tended to study spouses (Hooley, 1986; Hooley & Teasdale, 1989; Meuwly, Bodenmann, & Coyne, 2012) and have identified the role of criticism as particularly important within these relationships (Butzlaff & Hooley, 1998; Hooley, 2007). In studies of patients with dementia where the significant other may include the offspring of the patient, EOI appears to be very low indeed (Bledin, MacCarthy, Kuipers, & Woods, 1990; Orford, O’Reilly, & Goonatilleke, 1987; Tarrier et al., 2002). Thus, there may potentially be differences in the mechanisms of EE on patient outcomes, according to the nature of the relationship between the patient and the significant other. With respect to CFS/ME, it is likely that significant others’ experience of the condition may differ dependent on factors such as patient age and the nature of the relationship. Given the novel nature of the present study, and in line with the wider EE literature, significant others were identified by patients and not restricted to partner relationships only.

## Hypotheses

It was hypothesized that significant other high-EE (as defined by conventional criterion levels for critical comments and EOI) (Leff & Vaughn, 1985) would be associated with poorer patient outcomes, in comparison to significant other low-EE. Specifically, it was predicted that high levels of significant other EOI during the CFI would be associated with increased patient disability and fatigue severity. Furthermore, it was predicted that high levels of critical comments during the CFI would be associated with higher levels of patient depression. Given the previous associations between increased depression and poorer patient outcomes, it was also hypothesized that high levels of critical comments would be associated with increased fatigue severity, and that patient depression would mediate this association. No separate hypotheses were formulated with relation to hostility; it was expected that, if present, hostility would co-occur with high levels of critical comments.

Finally, it was hypothesized that there would be differences in the prevalence of EE observed within different patient-significant relationships. On the basis of previous literature it was expected that high levels of criticism would potentially be more characteristic of partner–patient dyads.

## Method

### Participants

To be eligible for participation patients had to have received a specialist diagnosis of CFS/ME, which was confirmed by a clinical study checklist based on the Oxford criteria for CFS/ME (Sharpe et al., 1991). In addition, patients had to have a willing significant other who had the most day-to-day involvement in the patients’ activities, and had a minimum of 10 hr face-to-face contact per week. Both participants were required to have sufficient fluency in English to complete the assessments. Any ongoing condition that may have impacted upon the significant others’ ability to complete the procedure, such as recovery from stroke, was set as an exclusion criterion; however, no participants were excluded during recruitment. Ethical approval was granted from a United Kingdom National Health Service (NHS) research ethics committee (11/NW/0198). Participants were recruited primarily from specialist NHS CFS/ME services; those patients who were newly inducted to the service or had recently begun specialist treatment were identified as potential participants. Thirty eight percent of patients approached within these services consented to being contacted by the researcher (*n* = 89), and of these, 52 consented to participating within the study (22% of those approached). Participants were also able to enter the study through self-referral as a result of advertisements with CFS/ME support organizations (*n* = 3). No incentives were offered in return for participation, and written informed consent was obtained from all participants. Participants aged 17 and over were recruited for the study as dyads; the final sample included 55 patients and their significant others. The patient sample ranged from 17 to 58 years old, with a mean age of 38 (12.25), and 91% of the sample were female (*n* = 50). The mean illness duration of the sample was 6.8 years upon entry into the study (range: 8 months to 25 years). Ninety three percent (*n* = 51) of the patient sample where White British. Mean (*SD*) scores on patient outcome measures at baseline and follow up are shown in Table 1. The age of significant others ranged from 19−72, with a mean age of 48 (12.87) years old, and 51% were female (*n* = 28). The majority of significant others were partners (*n* = 30, 55%), or parents (*n* = 20, 36%), while the remaining significant others included daughters (*n* = 3, 5%), sisters (*n* = 1, 2%), and close friends (*n* = 1, 2%). Ten patients (18%) did not live with their significant others, but in each case the significant other was the individual with the most daily contact with the patient.[Table-anchor tbl1]

### Procedure

Questionnaire measures were posted to each of the patients to complete before significant other interviews. All significant others were interviewed individually in their own or patients’ homes. All interviews were conducted confidentially, and audio-recorded. At 6 months after the baseline assessments, patients were sent a second copy of the fatigue, disability, and depression measures, to complete and return by post.

### Measures

#### Measures completed by the patient

Patient measures assessing CFS/ME outcomes were those widely reported within the literature (Dittner, Wessely, & Brown, 2004), and where possible, were those included in the United Kingdom CFS/ME national outcomes database (Collin et al., 2011). Reliability estimates for the current dataset are provided in Table 1.

##### Fatigue

###### Chalder fatigue questionnaire

Patient fatigue severity within the last month was assessed using the Chalder fatigue scale (Chalder et al., 1993). Eleven items assess both mental fatigue (i.e., “Do you have difficulty concentrating?”) and physical fatigue severity (i.e., “Do you lack energy?”); each item is rated on a 4-point scale from 0–3 (ranging from *better than usual* to *much worse than usual*). Individual scores are then summed to give a total fatigue score (0–33). The scale has been widely used (Cella & Chalder, 2010) and had excellent internal consistency at baseline and follow-up in the current sample.

###### Visual analogue scales of fatigue (VAS-F)

A visual analogue scale was also used to assess fatigue severity and energy levels experienced at the time of completion (Lee, Hicks, & Nino-Murcia, 1991). The scale consists of 18 items (13 measuring fatigue and 5 energy) each anchored with *not at all* to *extremely* and rated from 0–100; mean scores are calculated for each subscale. One item on the fatigue subscale was removed after piloting because of difficulties in comprehension in United Kingdom English (*not at all bushed* to *totally bushed*), resulting in a final 17-item scale. The measure has previously demonstrated good psychometric properties (Dittner et al., 2004).

##### Disability

###### The Short Form (36) Health Survey (SF-36)

Two widely used subscales examining physical functioning and bodily pain experienced within the last month were included (Ware & Sherbourne, 1992). Ten items assessed physical functioning; participants reported the extent to which they were able to perform typical daily activities (three possible response items: limited a lot; limited a little; not limited at all). Bodily pain was measured using two items assessing level of pain (rated on a 6-point scale from *none* to *very severe*) and pain interference (rated on a 5-point scale from *not at all* to *extremely*). Each scale is converted in to a score ranging from 0–100, higher scores indicating better patient functioning. The psychometric properties of the SF-36 have been extensively tested across countries (Gandek & Ware, 1998); the current data demonstrated reliability estimates in line with this.

##### Depression

###### Hospital Anxiety and Depression Scale (HADS)

All patients completed this scale designed to assess anxiety and depression during the previous week in patient populations (Zigmond & Snaith, 1983). It consists of 14 items that are rated on a 4-point scale from 0–3 (*not present* to *substantial*). Each subscale consists of 7 items that are summed to calculate a total score (ranging from 0–21), with higher scores indicating higher anxiety and depression. These subscales showed acceptable internal consistencies at baseline and follow-up within the current study (see Table 1). In the absence of hypotheses about anxiety, this subscale was not analyzed in the present study.

#### Measures completed by significant others

##### Expressed Emotion (EE)

###### Camberwell Family Interview (CFI)

All significant others took part in a semistructured interview used to assess levels of Expressed Emotion in patient populations (Vaughn & Leff, 1976). The interview was modified for relevance to CFS/ME, and included an additional focus on the period after the initial illness onset and preceding the current time, a section on illness management strategies and a section on the impact of the condition on daily life. The symptom section of the CFI was adapted to be relevant to CFS/ME. The interviews lasted ∼1 hr. The full interview may be obtained from the first author.

### Statistical Analysis

#### EE coding

All CFI interviews were conducted by the first author and rated using the conventional criteria (Leff & Vaughn, 1985). Critical comments are extracted when significant other utterances display strong tonal criticism or provide unambiguous evidence for disapproval of patient behaviors or characteristics. EOI is characterized by overidentification with the patient, or self-sacrificing, overprotective or emotionally exaggerated behaviors (Leff & Vaughn, 1985); see the supplemental material for examples. While an overall rating of High or Low EE (HEE and LEE) is made on the basis of scores on the critical comments and/or EOI and/or hostility subscales, to enable assessment of study hypotheses for this study, significant others were first classified on the basis of the critical comments subscale (designated HEE-C and LEE-C), and then reclassified according to their EOI status (HEE-EOI and LEE-EOI). Conventionally, HEE-C is assigned to significant others who make six or more critical comments, and HEE-EOI for those who demonstrate evidence for at least moderately high levels of EOI (equivalent to a score of ≥3 on the 0–5 scale) (Leff & Vaughn, 1985). An independent, EE-trained psychology doctoral student blind to the study hypotheses also second coded a selection of the interviews, and where there was disagreement on any rating, a third opinion was sought (*n* = 1). A random sample of these second rated interviews were selected to establish rating reliability (*n* = 9). Complete agreement was established for significant other EOI ratings, and acceptable agreement on the critical comments subscale (*r* = 0.89).

#### Data analysis strategy

SPSS version 20 was used to conduct all statistical analyses. Initial Kolmogorov–Smirnov tests were conducted to examine the distribution of the data; only VAS-fatigue and HADS subscales were normally distributed. Comparisons of patients who completed follow-up and those who did not were conducted for demographic variables, illness related variables and EE measures using χ^2^ (or Fisher’s exact test where appropriate) for categorical variables, and Mann–Whitney *U* tests for continuous variables. Demographic variables (age, gender) and illness related variables (illness duration, length of treatment) were correlated with both patient outcome variables (fatigue and disability) and predictor variables (EE) to identify any significant associations that may potentially inflate the relationship between predictor and outcome variables. These variables are henceforth referred to as confounding variables. The relationships between patient level of depression, outcome and EE variables was also assessed to determine the role of depression as a potential predictor variable. Comparisons of patient outcomes in HEE-C versus LEE-C, and HEE-EOI versus LEE-EOI dyads were performed using Mann–Whitney *U* tests. These comparisons were conducted at baseline and repeated at follow-up. Subsequently, regression analyses were conducted to assess EE-C and EE-EOI in combination with additional predictor variables in predicting patient outcomes at 6-month follow-up. Baseline outcomes were included within the models to control for previous level of functioning; further non-EE variables that were associated with outcomes at follow-up were identified as additional potential predictor variables. Mediation analyses were conducted using bootstrapping procedures to assess the mediational role of depression between HEE-C and fatigue severity. Finally, because of the heterogeneous nature of the significant other sample, exploratory analyses were conducted on the partner and parent subgroups. Comparisons between these groups were conducted using Fisher’s exact test for categorical variables, and Independent samples *t* tests for continuous variables (see Table 2).[Table-anchor tbl2]

## Results

### CFI Rated EE

Twenty significant others (36%) received a rating of overall high-EE. Very few critical comments were made overall within the sample; 26 significant others made no critical comments (47%). The mean number of critical comments was 1.91 (2.62); the median was 1 critical comment. Only 8 (15%) significant others made six or more critical comments, meeting the conventional threshold for a HEE-C rating. In addition, four of these critical significant others (7%) demonstrated evidence for hostile behaviors or attitudes. As expected, hostility always occurred in conjunction with HEE-C within the sample, and was therefore not analyzed separately. On the EOI scale, 14 (25%) significant others demonstrated no EOI (i.e., a rating of zero), and the median level was rated as ‘some EOI’ (equivalent to a score of 2) within the sample. Using the conventional criterion level of 3 or above, 16 significant others (29%) were classified as HEE-EOI, with 12 significant others (22%) achieving high-EE status based on evidence for HEE-EOI only.

### Preliminary Analyses

At baseline, patient levels of fatigue (VAS) were correlated with longer illness duration (*r*_s_ = .277, *p* = .045). Older patient age was significantly correlated with poorer physical functioning (*r*_s_ = −.448, *p* = .001). Longer treatment length was correlated with reduced patient anxiety (*r*_s_ = −.291, *p* = .040) and depression (*r*_s_ = −.352, *p* = .012). Additionally, patient level of depression (HADS) was significantly correlated with all patient fatigue outcomes (Chalder total fatigue, VAS fatigue and energy) and disability outcomes (SF36 physical functioning and bodily pain) (ranging from *r*_s_ = .189 to .473). Gender was not found to significantly relate to any of the outcome variables. All other correlations between potential confounding variables and patient outcome measures were nonsignificant. There were no significant associations between any of the potential confounding variables and the EE variables (critical comments and EOI). At follow up, patient depression (HADS) was significantly associated with all patient outcomes, and therefore baseline depression was included as a potential predictor variable in regression analyses, as were the baseline scores for the respective outcome measures to control for previous level of functioning.

### Expressed Emotion and Cross-Sectional Patient Outcomes

Contrary to predictions, there were no significant cross-sectional associations between significant other EE and patient outcomes (see Table 3).[Table-anchor tbl3]

### EE and Longitudinal Patient Outcomes

#### Six-month follow-up

Forty-four participants returned completed follow-up questionnaires (80% of baseline sample); of these, 41 were female participants (93%). Comparison analyses identified that there were no significant differences on demographic or illness related variables at baseline for those participants who completed follow-up compared with those who did not. Additionally, no significant differences were identified in significant other EE variables (overall EE, critical comments or EOI).

Comparisons of high- and low-EE groups (HEE-C vs. LEE-C and HEE-EOI vs. LEE-EOI) were conducted for patient outcomes at follow-up using Mann–Whitney *U* tests. In line with study predictions, patients whose significant other was rated as HEE-C at baseline had significantly higher levels of depression and fatigue at follow-up (see Table 3). As hypothesized, HEE-EOI at baseline was also associated with worse fatigue severity, but, contrary to predictions, not disability reported at follow-up (see Table 3).

#### Regression analyses

A series of regression analyses were conducted to examine if significant other EE independently predicted patient scores on outcome measures at follow-up. Predictor variables were selected as outlined in preliminary analyses.

As significant differences in fatigue severity were observed between groups for both HEE-C versus LEE-C and HEE-EOI versus LEE-EOI comparisons, multivariate analyses were conducted examining the individual impact of critical comments and EOI. Significant other HEE-C significantly predicted increased fatigue severity on the Chalder Fatigue scale (i.e., fatigue experienced within the last month) reported at follow-up (see Table 4). Once the other potential predictor variables were added to the model, HEE-C and depression remained significant predictors. When examining EOI only, HEE-EOI was the only significant predictor of fatigue severity at follow-up (see Table 4).[Table-anchor tbl4]

The above analyses were repeated for VAS fatigue scores (i.e., fatigue severity experienced at the point of follow-up); when entered alone, significant other HEE-EOI status predicted greater fatigue at follow-up. However, HEE-EOI became nonsignificant after the addition of patient depression (see Table 4). However, when examining the predictive validity of HEE-C only, both high critical comments and depression were predictive of greater fatigue severity in the final model.

Finally, HADS depression scale scores were examined (see Table 4). Having a significant other who was categorized as HEE-C, that is who made six or more critical comments during the CFI, was predictive of increased depression at follow-up. Baseline level of depression also significantly predicted depression reported at follow-up.

#### Relationships among patient fatigue severity, depression, and significant other critical comments

Having established that high levels of significant other criticism were predictive of increased levels of patient fatigue severity and depression, mediation was formally tested using the bootstrapping procedures outlined in Preacher and Hayes (2008). The basis for these analyses is that the indirect effect of HEE-C on the dependent variable (i.e., fatigue severity) is the product of the paths between HEE-C and mediator (i.e., depression), and between mediators and dependent variable. However, such indirect effects are not normally distributed, meaning that bootstrapping is necessary (Preacher & Hayes, 2008). Bootstrapping involves resampling random subsets of data to gain a nonparametric approximation of the sampling distribution of the product of the implementation intention-mediator and mediator-dependent variable paths. The analyses presented here are based on 1,000 resamples. The confidence intervals associated with the indirect effect of depression did not contain zero (95% CI = 2.01, 11.71). Thus, the effect of HEE-C on fatigue severity was significantly (*p* < .05) mediated by level of patient depression.

### Exploratory Analysis of Significant Other Subgroups

Patients who nominated their parent as their significant other were significantly younger than those with a partner; significant others within this group were also significantly more likely to be female (i.e., mothers compared with male partners). Significant differences were observed between partners and parents with respect to EE status; parents were significantly more likely to receive a high-EOI rating in comparison to partners. Furthermore, the four significant others who received a high-EE rating on the basis of evidence for both critical comments and EOI were all parents (see Table 2). No significant differences in significant other criticism were observed between these groups. Lack of statistical power prohibited any further exploration of potential differences between relationship subgroups.

## Discussion

This study examined the impact of significant other EE in a CFS/ME sample. The main findings demonstrate that significant other high-EE is associated with poorer longitudinal patient outcomes, particularly with respect to fatigue severity and depression. The predictive validity of high-EE is in line with the wider EE literature, which has demonstrated poorer illness and treatment outcomes for patients in high-EE dyads (Butzlaff & Hooley, 1998; Tarrier et al., 1999; Wearden, Tarrier, Barrowclough, et al., 2000).

In line with study hypotheses the role of the critical comments and EOI subscales were investigated separately. It was identified that high levels of critical comments were predictive of greater fatigue severity at follow-up compared to patients within low-EE dyads. Furthermore, on each of the fatigue outcome measures, both high critical comments and high levels of patient depression predicted greater fatigue at follow-up. Further analyses revealed that HEE-C was associated with, and predictive of, higher levels of patient depression at follow-up. It was hypothesized that the relationship between significant other HEE-C and poorer patient outcomes would be, at least, partially accounted for by increased levels of patient depression; the findings reported here demonstrated that depression did significantly mediate the relationship between HEE-C and fatigue severity. These findings appear to support the contention that significant other critical comments as measured by the CFI are theoretically linked with negative response styles; the associations reported here between HEE-C and patient outcomes are in line with the previous literature documenting an association between negative significant other responses and increased patient depression in CFS/ME (Romano et al., 2009; Schmaling et al., 2000). In addition, this evidence implies that these interpersonal processes may be more enduring than cross-sectional associations would suggest, or indeed that the effect of criticism is only observable when examining longitudinal associations within subjects. The findings provide further evidence for a potential underlying interpersonal mechanism; we speculate that highly critical EE may drive negative significant other responses. Finally, the association between HEE-C and depression may be particularly important clinically, since patient level of depressive symptoms have been previously found to be associated with poorer long-term outcomes after treatment in CFS/ME (Bentall et al., 2002), and recently was found to moderate the efficacy of pragmatic rehabilitation treatment in reducing patient fatigue severity (Wearden et al., 2012). A reduction in significant other HEE-C may therefore be efficacious in improving patient outcomes, particularly when engaging with specialist treatment, as in the current sample.

In contrast to high levels of critical comments, HEE-EOI was observed to predict fatigue severity only at follow-up. In this model, significant other EOI was found to be the only significant predictor of fatigue severity (whereas baseline fatigue and depression were nonsignificant). The results of this analysis suggest that EOI is potentially impacting upon fatigue severity through a separate mechanism other than increased depression, supporting the differentiation of these EE subscales acknowledged within the literature (Vasconcelos e Sa et al., 2013; Wearden, Tarrier, Barrowclough, et al., 2000). The finding that parents of patients were significantly more likely to be rated as HEE-EOI than partner significant others has been observed within other patient groups (Goldstein, Miklowitz, & Richards, 2002). Increased stress has often been cited as a potential mechanism through which EE may impact upon patient functional outcomes (Hooley, 2007), as previous evidence has suggested that interactions with high-EE significant others are experienced as more stressful by patients compared to interactions with low-EE significant others (Cutting, Aakre, & Docherty, 2006). It is possible that the beliefs and responses associated with EOI, such as overprotective and self-sacrificing behaviors, may be more or less acceptable and appropriate when considered within the context of these different groups (i.e., parents and partners). Lack of statistical power prevented further exploratory analyses of these processes within significant other subgroups within the current study; however, these outstanding questions surrounding the importance of the principle significant other relationship for patient outcomes in CFS/ME provide a clear direction for future research within this area. Additionally, it was proposed that high levels of EOI would be associated with increased patient disability, based upon the cross-sectional associations with solicitous responses reported in the literature (Romano et al., 2009; Schmaling et al., 2000). It is possible that this association was not observed within the current study because of the predominance of EOI in the parent significant other subgroup; previous research examining significant other responses within CFS/ME has focused solely on significant others involved in a romantic relationship with the patient. The lack of difference observed in the prevalence of high levels of criticism between parent and partner subgroups is of interest to the authors.

Although we have noted potential variations within the distribution of EE within significant other subgroups, as this is the first study to examine significant other EE in a CFS/ME sample, it is instructive to compare the distribution of high-EE in comparison to other patient populations. The number of critical comments made within the overall sample was very low, although the distribution was comparable to other samples where patients were experiencing a physical health problem (Wearden, Tarrier, & Davies, 2000). We had expected that critical comments may reflect significant other skepticism about illness legitimacy; however, many comments reflected significant other beliefs in a genuine illness (see supplemental material). Further work to understand the source of significant other criticism, when present, may be beneficial. Patients within the sample had been ill for a long duration before entering the study; many reported waiting a long time to engage with specialist services, and difficulties interacting with health care providers are commonly reported by patients (Larun & Malterud, 2007). The long duration of CFS/ME experienced by patients in the current sample before receiving specialist support may have reduced criticism within these close relationships (Blazquez & Alegre, 2013) out of increased pressure to formulate joint explanatory narratives of the condition (Brooks et al., 2013). However, despite the generally low levels of criticism within the sample, it is worth noting that when high levels of critical comments were present, patient outcomes at follow-up were poorer.

In comparison, a high proportion of significant others demonstrated high EOI; many of these receiving a high-EE rating on the basis of evidence for EOI only. Almost one quarter of significant others received a HEE-EOI only rating within the current study; other samples have identified much lower proportions of this classification across a range of conditions (Barrowclough, Johnston, & Tarrier, 1994; Tarrier et al., 2002; Wearden, Tarrier, & Davies, 2000). Evidence for self-sacrificing behaviors, intrusive overprotection and emotional display at interview were most commonly observed. The prevalence of HEE-EOI ratings may reflect the high number of parents involved as significant others within the study, but may also be due, in part, to the characteristics of the condition. High symptom severity and beliefs reflecting the potential beneficial effects of resting upon symptoms (Moss-Morris, 2005) may engender higher levels of self-sacrificing or overprotective behaviors from significant others. These significant other response styles may be in contrast to the general strategies recommended by the current United Kingdom management guidelines (NICE, 2007); therefore, inclusion of significant others in treatment programs may alleviate some of the impact of EE on patient outcomes (Brooks et al., 2013). Additionally, the high levels of emotional display reflect the difficulties experienced by this significant other group, and indicate that relatives of patients may also benefit from individual support or psycho-education in relation to the condition.

### Study Limitations

Although the majority of patients were newly inducted in to specialist treatment programs at the time of recruitment, no information regarding service use or engagement was available at follow-up. Therefore, the data has been analyzed using the available data and acknowledging the place of recruitment where possible. The low rates of EE, particularly critical comments, may lead to reduced power in detecting significant associations. This low prevalence of critical comments may be representative of the population of significant others of patients with CFS/ME but it may also reflect a self-selection bias; it is possible that highly critical significant others may have been less likely to participate, possibly because of beliefs about illness legitimacy. Future research should attempt to examine beliefs about the condition in association with significant other EE. As previously acknowledged, the heterogeneous nature of the current sample further limited statistical power for analyses examining the impact of EE within specific patient-significant other relationship types. A final additional limitation is the lack of independent measures of patient functioning; all patient outcome measures were assessed by patient self-report.

## Conclusions

The current study is the first to document the prevalence of significant other EE within a CFS/ME sample. The findings suggest that significant other high-EE is associated with poorer longitudinal patient fatigue outcomes, which are partially accounted for by increased levels of patient depression when high EE is as a result of high levels of significant other criticism. Therefore, the results provide the rationale for the integration of significant other psycho-education into current clinical interventions, to address significant other responses that may inadvertently contribute to symptom maintenance within this condition. To achieve this, future research should further clarify these interpersonal processes in CFS/ME by examining the associations between significant other relationship type and the development of EE. Research examining the associations between significant other beliefs and their emotional and behavioral responses in CFS/ME would also be beneficial. Finally, a reduction of significant other distress may also be a target for family oriented interventions.

## Supplementary Material

10.1037/hea0000086.supp

## Figures and Tables

**Table 1 tbl1:** Mean (*SD*) Scores for Patient Outcome Measures at Baseline and 6-Month Follow-Up

	Baseline		α	Follow-up		α
Outcome measure	Mean	*SD*		Mean	*SD*	
Fatigue						
Total fatigue (CF)	26.75	6.53	.938	19.73	9.65	.970
Fatigue (VAS)	60.59	17.75	.927	56.80	21.38	.947
Energy (VAS)	24.96	17.54	.909	31.55	22.77	.930
Disability						
Physical functioning (SF36)	34.73	24.73	.992	44.66	30.30	.949
Bodily pain (SF36)	40.13	26.90	.798	43.35	29.10	.928
Depression						
Depression (HADS)	9.46	3.99	.774	9.11	5.19	.884
*Note*. CF = Chalder fatigue Scale; VAS = Visual Analogue Scale; SF36 = Short Form (36) Health Survey; HADS = Hospital Anxiety and Depression Scale.						

**Table 2 tbl2:** Exploratory Analysis of Significant Other Subgroups

	Partner subgroup (*n* = 30)		Parent subgroup (*n* = 20)			
	Mean	*SD*	Mean	*SD*	*p*	d
Patient demographics						
Patient age (years)	43.27	8.62	28.85	10.50	**<.001**	1.50
Patient illness duration (years)	6.23	5.66	6.14	5.36	.954	.02
	*n*	%	*n*	%		*p*
Gender						.641
Female	27	90	19	95		
Male	3	10	1	5		
Living status						.096
With the SO	28	93	14	70		
Not with the SO	2	7	6	30		
Significant other variables						
Gender						**<.001**
Female	4	13	19	95		
Male	26	87	1	5		
Critical comments						.240
HEE-C	3	10	5	25		
LEE-C	27	90	15	75		
EOI						**.025**
HEE-EOI	5	17	10	50		
LEE-EOI	25	83	10	50		
HEE-C and EOI	0	0	4	20		**.021**
*Note*. HEE-C denotes high-EE as a result of critical comments. HEE-EOI denotes high-EE as a result of EOI. HEE-C and EOI refers to those significant others who received a high-EE rating on the basis of evidence for both critical comments and EOI. Fishers exact tests were conducted to compare characteristics of partner and parent SO subgroups; in these analyses, parents were coded as 0, partners as 1; males were coded as 0, females as 1; low-EE variables were coded as 0 and high-EE as 1. *p* < .05 is in boldface.						

**Table 3 tbl3:** Patient Mean Outcome Measures at Baseline and 6-Month Follow-Up by Low- and High-EE Subscales, and Significant Mann-Whitney U Tests

	Patient mean at baseline				Patient mean at follow-up				
	Low-EE	High-EE	U	*p*	Low-EE	High-EE	U	*p*	*r*
Critical comments									
CF total	26.5	29.38	235	.223	18.67	27.29	**194.5**	**.022**	.340
VAS fatigue	61.04	63.35	188.5	.914	54.03	73.03	**187**	**.045**	.303
VAS energy	26.03	17.85	126.5	.164	31.65	23.46	89.5	.236	−.181
HADS depression	9.5	10.13	194.5	.802	8.36	13.43	**201**	**.012**	.373
EOI									
CF total	26.84	21.29	272	.690	18.03	26.80	**254**	**.009**	.387
VAS fatigue	62.37	60.60	259.5	.524	51.86	74.51	**264**	**.003**	.429
VAS energy	24.77	24.39	296.5	.524	32.51	23.18	104.5	.082	−.262
SF36 Physical functioning	35.77	31.43	274.5	.606	46.36	39.50	135	.402	.130
SF36 Bodily Pain	40	40	273.5	.697	42.58	35	139.5	.786	.140
*Note*. *p* < .05 is in boldface.									

**Table 4 tbl4:** Summary of Hierarchical Regression Analysis for Variables Predicting Patient Scores on Outcome Measures at 6-Month Follow-Up (N = 44)

Variable	*R*^2^	Δ*R*^2^	B	*SE* B	β	*p*
Outcome: Chalder fatigue scale scores at 6-month follow-up						
Step 1	.115	.115				
High/low critical comments			8.62	3.73	.34	.**026**
Step 2	.292	.177				
High/low critical comments			7.92	3.44	.31	**.027**
Baseline fatigue			.04	.27	.05	.724
Baseline depression			1.90	.36	.40	**.009**
Outcome: VAS fatigue scale scores at 6-month follow-up						
Step 1	.109	.109				
High/low critical comments			19.04	8.49	.33	.**030**
Step 2	.398	.289				
High/low critical comments			16.16	7.21	.28	**.031**
Baseline fatigue			−.27	.18	−.21	.134
Baseline depression			3.37	.78	.60	**<.001**
Outcome: HADS depression scale scores at 6-month follow-up						
Step 1	.131	.131				
High/low critical comments			5.07	2.04	.36	.**017**
Step 2	.581	.450				
High/low critical comments			4.53	1.44	.323	**.003**
Baseline depression			.924	.141	.672	**<.001**
Outcome: Chalder fatigue scale scores at 6-month follow-up						
Step 1	.156	.156				
High/low EOI			8.77	3.18	.40	.**009**
Step 2	.277	.121				
High/low EOI			6.78	3.23	.31	.**043**
Baseline fatigue			.24	.27	.13	.391
Baseline depression			.74	.39	.29	.069
Outcome: VAS fatigue scale scores at 6-month follow-up						
Step 1	.202	.202				
High/low EOI			22.65	7.02	.45	.**002**
Step 2	.383	.181				
High/low EOI			13.75	6.92	.27	.054
Baseline fatigue			−.22	.19	−.17	.240
Baseline depression			2.87	.86	.51	**.002**
*Note*. *p* < .05 is in boldface.						
