# Supplementary material for: The Impact of Significant Other Expressed Emotion on Patient Outcomes in Chronic Fatigue Syndrome
Source: Health Psychol. 2014 Sep;33(9):1092–101. doi: 10.1037/hea0000086 (PMC4151798; doi:10.1037/hea0000086)
Supplement: Supplementary file 1 [file HEA20131428CFIcommentsSupplemental.docx]

**Appendix**

**Table 1:** Examples of statements similar to those extracted from the CFI interview: rated critical comments and evidence for EOI.

| Examples of significant other critical comments: |
| --- |
| - “I get frustrated with the fact that you know, sometimes we can’t go into town because it takes her a long time to get, you know, I'm ready to go with my coat on for 40 minutes like a kid who's waiting to go to the sweet shop, and its taking her all this time just to get dressed or just to do her hair or, it takes its toll actually...” - “Usually after tea she’s just laying on that couch, which annoys me because it’s not to be laid on” - “It can be 8 o'clock when I come home and then sometimes as soon as I walk through the door I start, I'll kick off because I'm thinking 'I've just done a 12 hour day and you've just got out of bed and you can't even tidy up a bit. I'm sure you've got the energy to do that!' and then it just kicks off because I can’t hold it anymore you know what I mean? At first it used to be like, ‘come on love’, I used to be so much more patient but I'm not anymore, I'm really not” - “I get very frustrated when she doesn't remember things, ‘cos I’ve got quite a good memory I think, and so there’s a lot of writing things down but even then she doesn't look at it, so I get frustrated by that” |
| Examples of significant other statements relevant for EOI: |
| - “I’ll come over and make her bed and make her a bit of breakfast and she usually just sits on the sofa until she’s strong enough to have a shower or a bath. I’ll pop to the shop and get her shopping, milk and fresh things that you know she needs. If it’s a good time, like at the moment, she gets up about lunchtime, you know about 12, and I’ll come and help her get ready and then she’ll go to work and immediately she comes home from work at about 5 or 6, I’ll come again just to help her in the evening, having made her tea and bring it round, and again usually she’s taken herself off to bed by 8o’clock” - “I worry constantly about how she’s feeling” - “What I would say is that his decision-making isn’t as good and what I would say is that he is forgetful and he gets things confused and I do put safeguards in place. I sound like a control-freak now but if you’re making important decisions, they have to be the right decisions so if it’s going to be an important decision which I can control I’ll ask him to check that out with me to make sure that’s okay.” - “The only time I really go out is to go down check on her mother, make sure she’s okay, you know what I mean, make sure she’s alright. I’ll go to my friend’s house for about an hour about twice a week, that’s it. I just don’t like leaving her in the house by herself, in case she feels worse and needs me to do something” - “In the end it was towards the end of last year I thought I can't cope with this anymore it's driving me insane you know, and I feel sorry for [patient], so sorry for him because he's the one got it, but it really affects everybody and this is why I've been upset, [patient]'s been upset, I'm gonna get upset again, because it just takes over” |
